# Coupling bimolecular PARylation biosensors with genetic screens to identify PARylation targets

**DOI:** 10.1038/s41467-018-04466-4

**Published:** 2018-05-22

**Authors:** Dragomir B. Krastev, Stephen J. Pettitt, James Campbell, Feifei Song, Barbara E. Tanos, Stoyno S. Stoynov, Alan Ashworth, Christopher J. Lord

**Affiliations:** 1 The CRUK Gene Function Laboratory and Breast Cancer Now Toby Robins Research Centre, The Institute of Cancer Research, London, SW3 6JB UK; 2 The Cancer Therapeutics Division, The Institute of Cancer Research, London, SW3 6JB UK; 3 The Institute of Molecular Biology, Bulgarian Academy of Sciences, 1113 Sofia, Bulgaria; 4 Present Address: UCSF Helen Diller Family Comprehensive Cancer Center, 1450 3rd Street, San Francisco, CA 94158 USA

## Abstract

Poly (ADP-ribose)ylation is a dynamic protein modification that regulates multiple cellular processes. Here, we describe a system for identifying and characterizing PARylation events that exploits the ability of a PBZ (PAR-binding zinc finger) protein domain to bind PAR with high-affinity. By linking PBZ domains to bimolecular fluorescent complementation biosensors, we developed fluorescent PAR biosensors that allow the detection of temporal and spatial PARylation events in live cells. Exploiting transposon-mediated recombination, we integrate the PAR biosensor en masse into thousands of protein coding genes in living cells. Using these PAR-biosensor “tagged” cells in a genetic screen we carry out a large-scale identification of PARylation targets. This identifies CTIF (CBP80/CBP20-dependent translation initiation factor) as a novel PARylation target of the tankyrase enzymes in the centrosomal region of cells, which plays a role in the distribution of the centrosomal satellites.

## Abstract (web summary)

Poly ADP-ribosylation (PARylation) is a highly dynamic post-translation protein modification, but most methods only detect stable PARylation events. Here the authors develop a split-GFP-based sensor for PARylation detection in live cells and use it to identify a new centrosomal PARylation target.

## Introduction

Poly ADP-ribosylation (PARylation) is a highly dynamic and reversible post-translation protein modification that is generated by a family of PAR polymerases (PARPs, ARTDs). The PARP superfamily encompasses 17 proteins, of which only PARP1, 2, and tankyrases (TNKS and TNKS2, also known as PARP5a and 5b) display a clear PARP activity^1^. The remaining family members are mono ADP-ribose transferases or lack enzymatic activity. PARP1, 2, and 3 are nuclear proteins involved in DNA damage responses (DDR)^2^, while tankyrases regulate a variety of cellular processes including telomere maintenance^3^, Wnt signaling^4^ and mitotic progression^5^. The role of PARP1, 2, and 3 in the DDR provided the rationale for the discovery and development of clinical PARP inhibitors. In addition, tankyrase inhibition can suppresses constitutive Wnt signaling^4^, which has led to the discovery of a series of small molecule TNKS/TNKS2 inhibitors^7^^8^. Given the burgeoning interest in the PARP superfamily enzymes as drug targets and their role as mediators of cellular signaling processes, identifying and characterizing the targets of these enzymes is critical.

A number of studies have identified PARylation targets en masse by isolating proteins that bind to either anti-PAR antibodies or PAR-binding protein domains and identifying these by mass-spectrometry^9^. PARylation is often a transient modification, therefore, some studies have used exposure to DNA-damaging agents to enhance DNA damage-dependent PARylation, or suppression of PAR glycohydrase (PARG) to prevent PAR degradation^10^. An additional complication of such studies is that PARylation is often induced on non-specific targets during in vitro cell lysis^9, 11^. Hence, additional approaches to detect and characterize PARylation targets are required.

In this study, we describe a system for identifying and characterizing PARylation events that exploits the ability of PBZ (PAR-binding zinc finger) domains to bind PAR with high-affinity. By linking PBZ domains to bimolecular fluorescent complementation (BiFC) biosensors, we developed fluorescent PAR biosensors that allow the detection and localization of PARylation events in live cells. Finally, by exploiting transposon-mediated recombination, we integrated these PAR biosensors en masse into thousands of protein coding genes in living cells. Using these PAR-biosensor “tagged” cells in a genetic screen facilitates the large-scale identification of PARylation targets. Using this approach, we show that CTIF (CBP80/CBP20-dependent translation initiation factor) is a target of PARylation by tankyrases at centrosomes and plays a role in the distribution of the centrosomal satellites.

## Results

### PAR-binding domains as high-affinity cellular biosensors

**Fig. 1:**
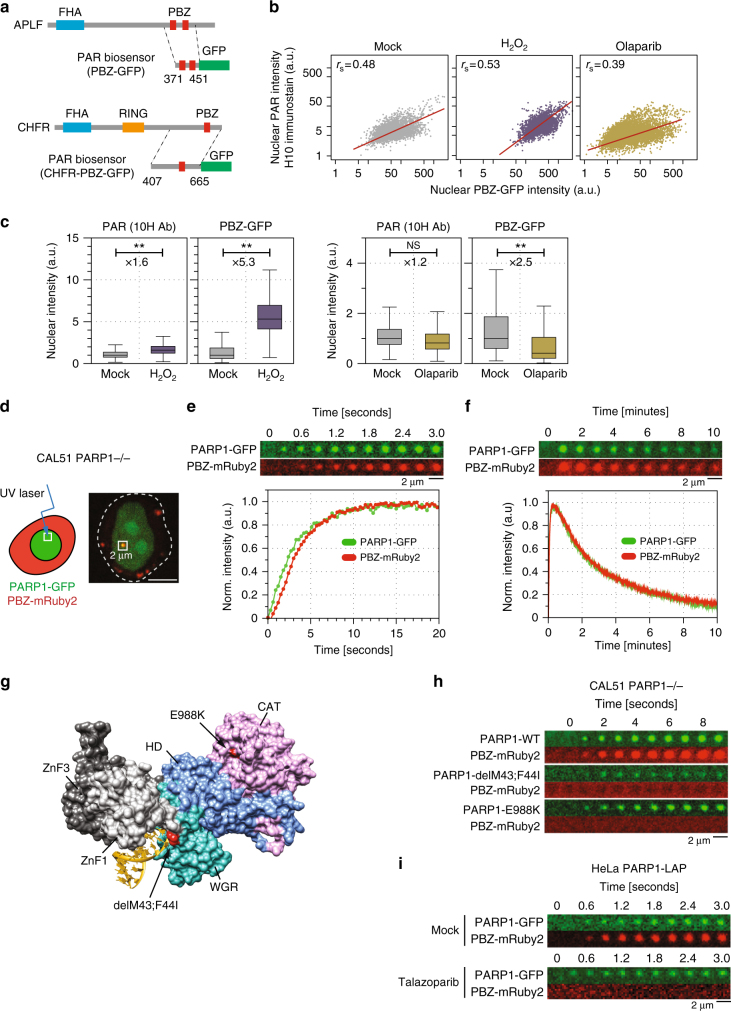
PAR biosensors detect cellular PARylation. **a** PAR-binding biosensors derived from APLF and CHFR. The PAR-binding PBZ domains were fused to full-length GFP, generating PAR biosensors PBZ-GFP and CHFR-PBZ-GFP, respectively. When CHFR-PBZ biosensor is used it is explicitly annotated; in all other cases the APLF PBZ biosensor is used. **b**, **c** PAR-binding biosensor signal correlates with PAR immunodetection but has a greater dynamic range. HeLa cells expressing PBZ-GFP were exposed to 1 mM H_2_O_2_ or 1 μM olaparib; GFP signal and PAR (10 H anti-PAR antibody) were monitored 10 min after exposure. **b** Scatter plots of the PAR biosensor intensity correlate with the PAR Ab detection. Intensities were measure in > 1000 cells per condition. **c** Fold change in median signal in H_2_O_2_ vs. mock, and olaparib vs. mock are shown for both PAR detection approaches (exemplary images are shown in Supplementary Fig. 1A). NS–not significant, box plot shows quartiles, Student’s *t*-test **p-values < 0.01. **d**, **f** Kinetics of PARylation at sites of DNA damage. *PARP1* null CAL51 cells (CAL51 *PARP1*^–/–^) were transfected with PARP1-GFP and PBZ-mRuby2 and exposed to localized (micro)irradiation as shown in **d**. After microirradiation, PARP1 and PAR localization were monitored over time. A microirradiated cell is shown; the area that is shown on the subsequent kymographs is annotated with a white box with a 2 µm side. Scale bar represents 5 µm. **e** Kymograph (top) and graph (bottom) of PARP1 and PBZ-mRuby2 0–3 s after microirradiation. **f** as per **e** but 0–10 min after microirradiation. Each graph shows average signals from > 10 cells; scale bar represents a distance of 2 µm. **g**, **h** A PAR biosensor detects loss of PAR at microirradiated sites caused by PARP1 mutations. **g** PARP1 bound to a double strand break (4OQB) with indicated: p.[43delM;44 F > I] and E988K mutations in red. **h** Kymographs are shown from microirradiated CAL51 *PARP1*^–/–^ cells expressing PBZ-mRuby2 and either wild-type PARP1-GFP, PARP1-p.[43delM;44 F > I]-GFP, or PARP1-E988K-GFP. **i** The clinical PARP inhibitor talazoparib reduces PAR levels at sites of microirradiation. Kymographs of HeLa cells with a PARP1-GFP containing bacterial artificial chromosome (PARP1-LAP) and PBZ-mRuby2 were exposed to 100 nM talazoparib for 1 h prior to microirradiation

We aimed to develop a set of PAR-biosensors that could detect PARylation events in living cells. To do this, we exploited the PAR-binding ability of PBZ (PAR-binding zinc finger) domains derived from either APLF (aprataxin PNK-like factor) or CHFR (checkpoint protein with FHA and RING domains) to bind PAR with high-affinity^12^. Although several other PAR-binding domains exist (such as macro and WWE domains), we selected PBZ domains for the development of biosensors for the following reasons: (i) their well-defined structure with the possibility to engineer precise point mutations that abolish PAR binding^12^, and (ii) their intermediate PAR-binding affinity (weaker compared to macrodomains), which allows reversible binding (this is confirmed below), thus minimizing the possibility of artefactual PAR stabilization and interference with endogenous PARylation-dependent processes. We fused the coding sequence of the APLF or CHFR-PBZ domain to that of green fluorescent protein (GFP), generating PAR biosensors (Fig. 1a; from here onwards PBZ refers to the APLF domain and CHFR-PBZ will be explicitly written when it is used). We then compared the ability of PAR biosensors to detect DNA damage-induced PAR, when compared to PAR immunodetection with a commonly used anti-PAR antibody (10H). To elicit DNA damage-induced PARylation, we exposed HeLa cells to H_2_O_2_; to reduce PAR, we exposed cells to the PARP1/2 inhibitor olaparib. In untreated cells, cells exposed to H_2_O_2_, or cells exposed to olaparib (Fig. 1b), the antibody and the biosensor signals were correlated (Spearman’s rank correlation, *r*_s_ = 0.48, 0.53, and 0.39 respectively) suggesting that the biosensor signal recapitulated the detection of PARylation shown by immunodetection (exemplary images are shown in Supplementary Fig. 1a). H_2_O_2_ exposure caused a close to twofold increase in 10H PAR signal compared to the basal state, while olaparib treatment did not lead to any significant decrease in PAR signal (Fig. 1c), consistent with the notion that the 10H antibody predominately recognizes long damage-induced PAR chains, but fails to detect endogenous PARylation. In contrast, the PAR biosensor revealed a broader dynamic range; H_2_O_2_ exposure caused an above fivefold increase in the nuclear GFP intensity (Fig. 1c), while olaparib treatment led to a twofold decrease, resulting in a 12-fold difference between the absence and damage-induced PAR levels.

We also assessed the ability of a PAR biosensor to monitor temporal changes in PARylation. To do this, we used PBZ-mRuby2 biosensor alongside a PARP1-GFP expression construct; this allowed us to temporally co-monitor PARP1 and PAR localization on ultraviolet microirradiated regions of cells. To eliminate any potential interference from endogenous PARP1, we carried out these experiments in *PARP1*^–/–^ cells (Methods; Fig. 1d). Localized laser microirradiation led to a rapid localization of PARP1 to the site of DNA damage (within 200 ms), followed 300 ms later by localization of PAR at the same site (Fig. 1e). After 30–60 s, both the PARP1-GFP and PBZ-mRuby2 signals were reduced in a co-ordinated fashion (Fig. 1f), likely reflecting reduction of PARP1 localization and activity after the initial stages of DNA repair. The binding of the biosensor to the microirradiated site was rapid and reversible as shown by FRAP experiments (Supplementary Fig. 1c). To confirm the specificity of this effect, we used two mutant PARP1-GFP fusions Fig. 1g): a DNA-binding deficient mutant of PARP1 with mutations of residues 43 and 44 that disrupt the ZnF1 domain (PARP1-p.[43delM;44 F > I]^13^); or PARP1 with an E988K mutation that impairs catalytic activity. The PBZ-mRuby2 sensor signal at microirradiated regions was entirely dependent upon wild-type PARP1 (Fig. 1h). Exposure of HeLa cells to the clinical PARP inhibitor talazoparib also abolished the PBZ-mRuby2 sensor signal (Fig. 1i). Importantly, in this experiment PARP1-GFP was expressed at endogenous levels from a bacterial artificial chromosome (BAC) showing that PARP1 overexpression does not alter the behavior of the biosensor. Furthermore, using HeLa cells without any additional PARP1 expression, we assessed the behavior of PBZ-mRuby2 and CHFR-PBZ-GFP (Supplementary Fig. 1b). Both biosensors showed identical kinetics, confirming that the observed results are due to the dynamics of PAR modification rather than the specificities of the PBZ domain used. Taken together, this data suggested that the PAR biosensors we developed exhibited high sensitivity and PAR-dependent behavior and could be used to dynamically monitor the amount of cellular PARylation.

### Development and validation of BiFC biosensors of PARylation

**Fig. 2:**
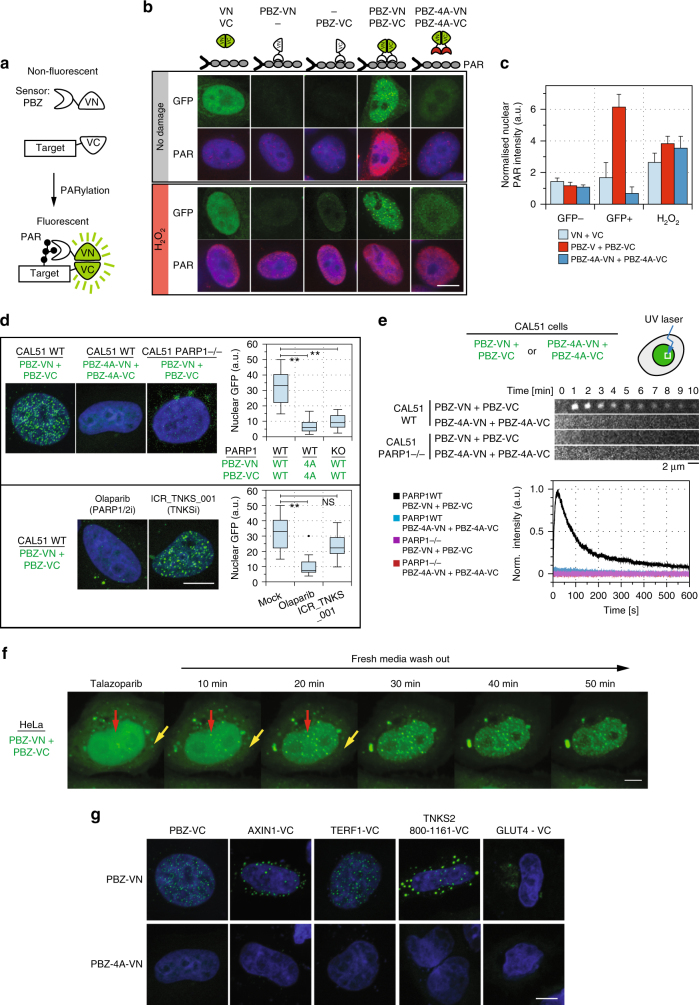
Bimolecular fluorescent complementation (BiFC) PAR biosensors detect cellular PARylation. **a** BiFC PAR biosensor-PBZ coding sequence was fused to the N-terminus of Venus (VN) (PBZ-VN); the C-terminus of Venus (VC) was fused to query protein (protein-VC). PARylation of the query protein leads to VC–VN interaction and restoration of a fluorescent GFP. **b** HeLa cells were transfected with constructs expressing: VN + VC, PBZ-VN, PBZ-VC, PBZ-VN + PBZ-VC or PBZ-4A-VN + PBZ-4A-VC (4A constructs lack PAR binding). Cells were mock-treated or exposed to 1 mM H_2_O_2_ and stained with PAR-binding reagent (Millipore). GFP nuclear foci are observed only in the PBZ-VN + PBZ-VC, and are marked by PAR-binder staining. **c** A quantification of PAR nuclear intensity as shown in **b**. GFP + and GFP-represents the PAR intensity of sensor-transfected or not-transfected cells. PBZ-VN + PBZ-VC expression leads to PAR accumulation in the absence of damage. Mean and standard deviations are shown for > 20 nuclei. **d** Confocal images of *PARP1* wild-type or *PARP1*^–/–^ CAL51 cells expressing, PBZ-VN + PBZ-VC or PBZ-4A-VN + PBZ-4A-VC. PBZ-4A. Loss of PARP1 or PARP1 inhibition (olaparib) ablated the formation of nuclear GFP foci, while the tankyrase inhibitor (ICR-TNKS-001) did not; mean nuclear GFP intensity from > 20 nuclei; box plot shows quartiles, **Student’s *t*-test *p*-values < 0.01. **e** Kinetics of PARylation at microirradiation sites. Kymographs and graphs of the GFP signal in *PARP1* wild-type or *PARP1*^–/–^ CAL51 cells with either PBZ-VN + PBZ-VC sensors or PBZ-4A-VN + PBZ-4A-VC sensors. **f** The PARP inhibitor talazoparib modulates PAR foci. HeLa cells, expressing PBZ-VN + PBZ-VC, were exposed to 100 nM talazoparib overnight; the cells were washed in drug-free media and imaged. After removal of talazoaprib, biosensor signal in the cytoplasm (yellow arrow) reduced; while the frequency of GFP + nuclear foci (red arrows) increased. **g** PAR Biosensor detects nuclear/cytoplasmic localization patterns of known PARylated proteins. HeLa cells were cotransfected with PBZ-VN or PBZ-4A-VN plus AXIN-VC, TERF1-VC, TNKS2_800-1161-VC or GLUT4-VC. PBZ-VN transfected cells revealed protein-specific localization pattern, while PBZ-4A-VN transfected cells did not. Scale bars represent 5 µm

The identification of PARylation targets via biochemical purification is confounded by the artefactual loss and gain of PARylation events during cell lysis^9^. As the PAR biosensors described above provided the ability to detect PARylation events in living cells, rather than in cell lysates, we assessed whether we could modify these so that they could be used in genetic screens to identify novel PARylation targets. To do this, we needed to solve two issues: (i) to design PAR biosensor systems that monitored the PARylation state of specific proteins, rather than the total amount of cellular PAR; and (ii) to design PAR biosensors that could stabilize what might otherwise be relatively transient PARylation events. With these issues in mind, we designed a bimolecular fluorescent complementation (BiFC, “split-GFP”) approach, shown schematically in Fig. 2a. In BiFC approaches^14^, two non-fluorescent halves of a GFP molecule are expressed as fusion proteins with two query proteins; for example, the C-terminus of Venus GFP (VC) is fused to a query protein and the N-terminus of Venus GFP (VN) is fused to the PBZ domain (Fig. 2a). Hence, this would allow us to detect PARylation events by reconstituting a functional GFP molecule, when the query protein is PARylated. One advantage of such a system would be that while the PARylated state of some proteins might have biochemical half-lives in the range of seconds to minutes^15, 16^, the half-life of Venus GFP, once formed by VC-VN complementation, is in the range of hours^14^, potentially stabilizing these events.

To test this approach, we generated PBZ-VN and PBZ-VC biosensors. Simultaneous introduction of these probes into HeLa cells in the absence of exogenous DNA damage generated a characteristic pattern of multiple GFP-positive nuclear foci (Fig. 2b). When used in isolation, neither PBZ-VN nor PBZ-VC generated a detectable GFP signal. This suggested that the BiFC approach, compared to PBZ-GFP, might provide a more sensitive approach to monitoring PARylation events in situ.

**Fig. 3:**
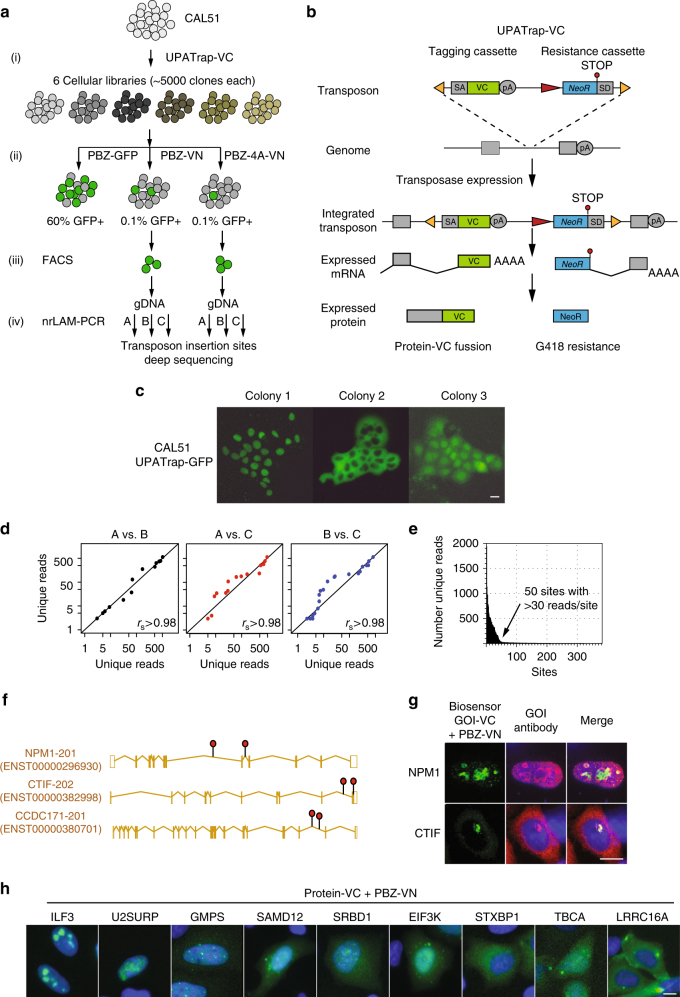
A transposon-based biosensor screen to identify PARylated proteins. **a** A genetic screen to identify PARylation events. (i) UPATrap-VC was introduced into CAL51 cells, generating six tagged cell libraries (5000 clones each). (ii) Either PBZ-GF, PBZ-VN, or PBZ-4A-VN biosensors were introduced into each library and GFP-positive cells were isolated (iii); PBZ-GFP showed 60% GFP-positive cells, while the PBZ-VN constructs showed 0.1% GFP-positive cells. (iv) Genomic DNA was isolated from GFP-positive cells and UPATrap-VC integration sites identified by non-restrictive linear amplification PCR (nrLAM-PCR) followed by deep-sequencing. Each gDNA sample was amplified and sequenced in three independent reactions (A, B, C). **b** A schematic of the transposon-mediated VC tagging, when an UPATrap-VC transposon is introduced into genes. Yellow triangles *Tol2* transposon repeats, SA splicing acceptor, SD splicing donor, IRES internal ribosome entry site, NeoR G418-resistance gene, pA polyAdenylation signal. Integration of UPATrap-VC into genes results in the production of protein-VC fusion proteins and NeoR protein. **c** Integration of a full-length GFP version of UPATrap (UPATrap-GFP) generates specific localization pattern in different GFP-positive colonies. This suggests that the transposon has captured and generated in-frame protein-VC fusion in a specific gene. **d** nrLAM-PCR and deep-sequencing from independent reactions is highly reproducible. Scatter plots are shown illustrating the correlation between unique read depth from three replica nrLAM-PCR and sequencing reactions (A, B, and C); Spearman’s rank correlation > 0.98. **e** Distribution of sequencing depth across all libraries in the screen. Approximately 50 genomic sites were represented by a unique read depth of > 30 reads (see detailed description in methods). **f** A schematic representation of the three genes identified with two, independent, transposon integration sites (indicated by red circles). **g** Biosensor signal obtained by the expression of full-length NPM1-VC and CTIF-VC in combination with PNZ-VN. The cells were co-stained with antibodies, recognizing the endogenous NPM1 and CTIF, respectively. **h** PARylation biosensor screen detects “hits” with different subcellular localization. Confocal imaging of VC-VN GFP signal for 11 genes identified in the screen are shown. NPM1 is a known PARylation target. Scale bars represent 5 µm

**Fig. 4:**
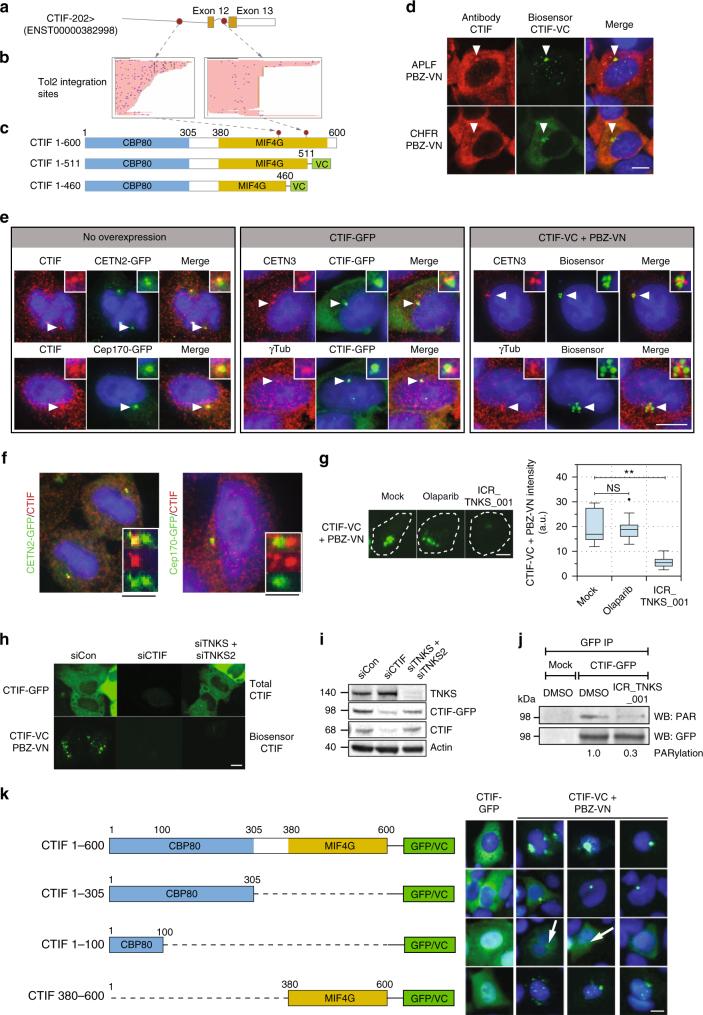
CTIF is PARylated in a TNKS-dependent manner. A schematic of the *CTIF* 3′ end with Tol2 integration sites (red circles) (**a**), nrLAM-PCR products (**b**) and generated CTIF-VC fusion proteins (**c**) identified in the screen. **d** CTIF biosensor is localizes to the centrosomal area of the cell (white arrowheads). HeLa cells were transfected with CTIF-VC + PBZ-VN or CHFR-PBZ-VN and immunostained with an anti-CTIF antibody. **e** CTIF localizes with centrosomal markers. HeLa cells were transfected with centrosome markers CETN2-GFP or Cep170-GFP, and co-stained with anti-CTIF antibody. Cells expressing either CTIF-GFP or (CTIF-VC + PBZ-VN) were co-stained with anti-CETN3 or anti-γTubulin. CTIF-GFP is broadly distributed in the cells, while the CTIF biosensor is surrounding the centrosome. White arrowheads indicate the area that is shown in insets with 2 µm side. **f** CTIF localizes to the daughter centriole. HeLa cells were transfected with CETN2-GFP (marks both centrioles) or Cep170-GFP (marks the mother centriole), and stained with anti-CTIF antibody. **g** Tankyrase inhibition reduces CTIF biosensor signal. HeLa cells, expressing CTIF-VC + PBZ-VN, were exposed to olaparib or ICR-TNKS-001. Quantification of the GFP signal over > 20 cells in three independent experiments is shown, NS–not significant, box plot shows quartiles, **Student’s *t*-test *p*-values < 0.01. **h** Tankyrase depletion reduces CTIF biosensor signal, as in **g** TNKS + TNKS2 siRNA did not reduce CTIF expression (CTIF-GFP). The efficiency of depletion is shown on the western blot in **i**. **j** CTIF is a direct PARylation target. CTIF-GFP was immunoprecipitated with anti-GFP antibody and immunoblotted with anti-PAR (10 H) antibody. Uncropped blots are shown in Supplementary Fig. 4F. **k** CTIF has a N-terminal CBP80 (AA 1-305) and a C-terminal MIF4G (AA 380–600) domain. A deletion series was fused to either full-length GFP or VC sequence. CAL51 cells, expressing GFP-fused variants showed broad cytoplasmic distribution, while the VC-fused ones showed localized patterns. Centrosome signal was observed with the full-length CTIF, and with the N-terminal CBP80 domain, down to the 100 most N-terminal amino acids (white arrows). All constructs were expressed at a similar protein level (Supplementary Fig. 4g). Scale bars represent 5 µm

**Fig. 5:**
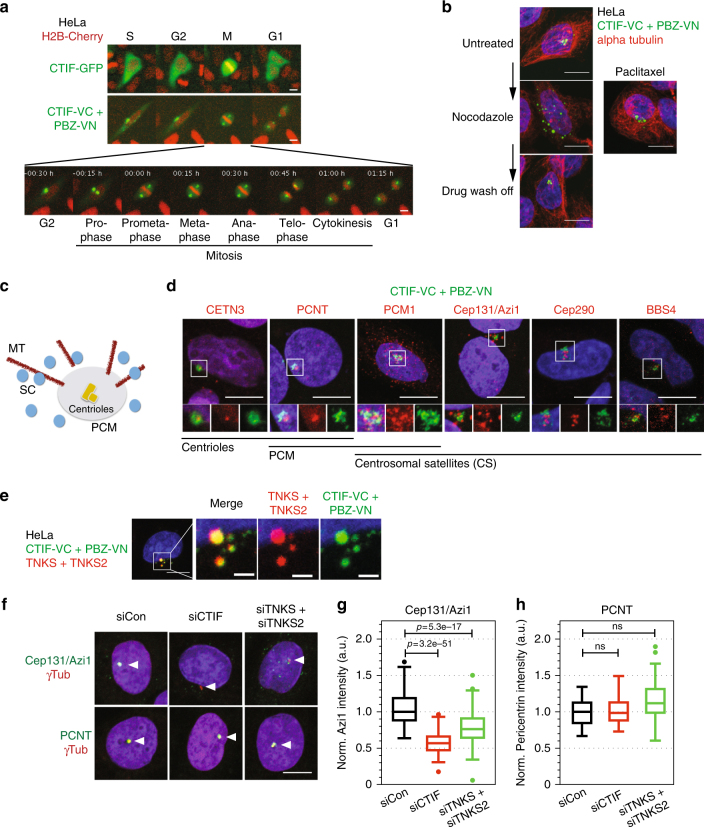
CTIF modulates centrosomal satellites. **a** CTIF biosensor localizes and segregates with the centrosome. Live cell imaging of H2B-cherry HeLa cells expressing either CTIF-GFP or CTIF-VC + PBZ-VN showed that CTIF-GFP is distributed throughout the cytoplasm during M phase (top images), while CTIF-VC + PBZ-VN signal (lower images) segregated with the centrosome during mitosis. Detailed time series are shown in Supplementary Fig. 5a. **b** CTIF biosensor localization is microtubule-dependent. HeLa cells expressing CTIF-VC + PBZ-VN were exposed to either nocodazole or paclitaxel. Nocodazole caused a reversible dispersal of the signal while the microtubule-stabilizing drug paclitaxel did not. **c** Schematic illustrating the centrosome with a central pair of centrioles, surrounded by pericentrosomal material (PCM), microtubules (MT) and centrosomal satellites (CS). **d** Co-localization of CTIF biosensor and various centrosomal markers showed that PARylated CTIF is peripheral to inner centrosomal markers (CETN3 and PCNT) and PCM (PCNT and PCM1), while it is localized in the area occupied by the centrosomal satellites (Cep131/Azi1, Cep290 and BBS4); insets are with 2 µm sides. **e** CTIF PARylation biosensor co-localizes with tankyrase. HeLa cells transfected with CTIF-VC + PBZ-VN were immunostained for endogenous tankyrase; scale bars in the zoomed images represent 1 μm. **f** CTIF depletion affects the centrosomal satellites, but not the core centrosome. HeLa cells were depleted by *CTIF* or tankyrase (*TNKS* + *TNKS2*) siRNA and immunostained for various centrosomal markers. Images show staining for the centrosomal satellite marker Cep131/Azi1 and for the core centrosome marker PCNT together with centrosomal marker γTubulin. Images for all the other analyzed markers are shown in Supplementary Fig. 5b and full summary is provided in Table 2. The average intensity of the signal at the centrosomal area was quantified in *n* = 150 nuclei and normalized to the median of the mock-transfected cells. Box plot shows that CTIF depletion, and to a lower extend tankyrase depletion, leads to the dispersal of the centrosomal satellite (Cep131/Azi1 quantified in **g**), while not affecting the core centrosome (PCNT quantified in **h**); box plot shows quartiles, *p*-values were calculated by two-sided Student’s *t*-test. Scale bars represent 5 µm

To assess the specificity of the BiFC approach, we generated sensors that contained four cysteine to alanine mutations within PBZ (equivalent to APLF amino-acid positions p.C379A, C385A, C421A, and C427A) known to abolish PAR binding^12^; we termed these probes as PBZ-4A-VN, and PBZ-4A-VC. The introduction of these mutations abolished the formation of GFP-positive nuclear foci (Fig. 2b). Co-staining of the cells with a PAR-binding reagent (MABE1031, Millipore; Fig. 2b, red) showed that only the co-expression of the wild-type PBZ probes led to PAR stabilization, while this was not the case for single probes or the PBZ-4A co-expression (quantification of this effect is shown in Fig. 2c). The split-GFP PBZ biosensor showed around sixfold increase in PARylation, while a PARylation inducing treatment (10 min of 1 mM H_2_O_2_) showed 3–4 fold induction. Importantly, in all the subsequent experiments shown in Figs. 3, 4, and 5 to detect PARylation on target proteins, we express only one PBZ construct (typically PNZ-VN), which does not lead to PAR stabilization on its own.

We introduced these sensors into *PARP1*^–/–^ CAL51 cells and found that the absence of PARP1 prevented the detection of both GFP-positive nuclear foci and localized microirradiation PAR/GFP signal (Fig. 2d, e). This observation was, therefore, consistent with the central role of PARP1 as a nuclear PARP enzyme associated with the response to DNA damage^17^. Consistent with this observation, we found that the PARP1/2 inhibitor olaparib reduced PBZ-VC + PBZ-VN nuclear foci, while a potent PARP5A/B (Tankyrase) inhibitor ICR-TNKS-001 (ref.^8^) did not (Fig. 2d).

Microirradiation experiments demonstrated that PBZ-VC + PBZ-VN could also monitor the temporal increase and then decrease in PARylation associated with microirradiation (Fig. 2e). In this case, only the wild-type PBZ constructs, transfected in PARP1 wild-type cells, showed efficient recruitment, showing the dependence of this on the presence of PARP1. We believe that the kinetics of recruitment in these experiments reflect the properties of recruitment of the split-GFP PBZ probes rather than in situ assembly of the GFP molecules. The formation of a mature GFP fluorophore in situ of assembly is typically observed in 10 min (as discussed in Fig. 2f)^18^, hence the signal detection in the range seconds is likely a reflection of the recruitment of a fraction of pre-assembled GFP molecules. The PBZ-4A-VN + PBZ-4A-VC sensors did not detect this dynamic PAR signal (Fig. 2e). In addition, the accumulation and dissipation of PAR was also not detected when PBZ-VN + PBZ-VC sensors were used in *PARP1*^–/–^ cells, suggesting once more that this PAR signal was PARP1 dependent (Fig. 2e). These kinetics were identical to those observed with the PBZ-GFP sensor (Fig. 1f) suggesting that the fusion into the BiFC does not alter the recognition and recruitment to PARP1-mediated PARylation sites.

Using PBZ-VN + PBZ-VC sensors to detect the temporal response to PARP1/2 inhibitor exposure (talazoparib) GFP foci were found in the cytoplasm (Fig. 2f, yellow arrows) but were less frequent in the nucleus; when talazoparib was removed from the tissue culture media, nuclear GFP foci reformed within minutes (Fig. 2f, red arrows), while the frequency and intensity of cytoplasmic PAR foci was reduced. One explanation for this effect might be that the cytoplasmic GFP foci represented non-PARP1/2-mediated PARylation events (by PARP enzymes such as tankyrases, PARP4 and/or PARP10/14/16, which are not inhibited under these conditions), while nuclear GFP foci represented PARP1/2-mediated PARylation; upon the removal of talazoparib, there is a rapid shift of PARylation from being mostly cytoplasmic to mostly nuclear due to the reactivation of PARP1. It should be noted that the possibility that talazoparib induces cytoplasmic stress granules, which are enriched in PAR, cannot be excluded as an alternative explanation. The appearance of the nuclear foci coincides with the time of GFP fluorophore maturation in situ of the split-GFP assembly, which is around 10 min^18^, and it is unlikely to be due to sensor diffusion as this is a rather fast process (as assessed in Supplementary Fig. 1c).

Lastly, we assessed whether the BiFC sensors could identify known PARylation events associated with PARP superfamily enzymes other than PARP1/2. To do this, we generated VC-fused sensors for four “query” protein PARylation targets of Tankyrase (PARP5A/B, TNKS/TNKS2): AXIN^4^; TERF1 (aka TRF1)^3^; TNKS2 itself (amino acids 800–1161, encompassing the SAM and CAT domains of the protein)^4^; and GLUT4^19^. These query protein-VC sensors were introduced into HeLa cells alongside either PBZ-VN or PBZ-4A-VN (Fig. 2g). In each case, combining the PBZ-VN sensor with a query protein-VC revealed a detectable fluorescent signal that had the expected cellular localization pattern (cytoplasmic degradosomes for AXIN and TNKS2, nuclear for TERF1, perinuclear and golgi-like for GLUT4, Fig. 2f). In contrast, the PBZ-4A-VN biosensor generated only a marginally detectable fluorescent signal. We validated these signal by immunostaining with antibodies recognizing the endogenously expressed proteins (TERF1 and GLUT4 shown in Supplementary Fig. 2b, c), which revealed partial or complete overlap of both signals. This suggests that the biosensor recognizes, in situ, only the PARylated fraction of the protein. It is particularly clear in the case of TERF1 (Supplementary Fig. 2b), which the antibody recognized throughout the nucleoplasm with some enrichment at the telomeres, while the biosensor signal is predominantly telomeric, which is where TRF1 is being PARylated^3^. Furthermore, CHFR-PBZ-VN biosensor showed virtually identical pattern to PBZ-VN, strengthening these observations.

Taken together, we concluded that these BiFC sensors demonstrated sufficient specificity for the PARylation state of a series of proteins and could be used to identify novel PARylation targets in situ, as described below.

### A BiFC-based screen identifies novel PARylation targets

Using the BiFC sensors, we designed a genetic screening system aimed at identifying novel PARylation targets. The screening approach involved (Fig. 3a): (i) generating cellular libraries where each cell contained a VC-coding sequence integrated into an endogenous gene by the use of a gene trap transposon^20^; (ii) introducing a PBZ-VN sensor into this cell library; (iii) selecting cells with VC-VN detectable PARylation events by FACS GFP-positive cells; and (iv) identifying the VC-containing gene by deep-sequencing of genomic DNA flanking the transposon.

We relied upon the ability to integrate an in-frame VC-coding sequence into multiple endogenous genes in any given cell population. This generated a population of cells where the expression of query gene-VC fusion was largely controlled by the native promoter and enhancer DNA sequences, rather than driving the transcription of query protein-VC fusions from a plasmid-based cDNA. Gene trap transposons provide an effective way of generating such gene fusion events within endogenous genes and so we adapted a Tol2-based UPATrap vector to encompass a VC-coding sequence (a schematic of how the gene-trapping events occur is shown on Fig. 3b). Because Tol2 is a sequence-independent cut-and-paste transposon^21^, only one of the two alleles of a gene is likely to be trapped, which implies that there would not be detrimental cellular effects because of the second allele. We used the previously published UPATrap^21^, which contains two functional DNA cassettes: (i) at the 5′-end, a promoterless splice acceptor (SA)-IRES-GFP-polyA sequence; and (ii) at the 3′-end, a promoter-driven *NeoR* (G418 resistance) coding gene with a 3′ splice donor sequence (SD). We modified UPATrap in three significant ways to allow the generation of protein fragments fused to VC: (Supplementary Fig. 3a): (1) by replacing the IRES-GFP sequences with a VC-coding sequence, generating a SA-VC-polyA cassette; (2) by generating three different open reading frames of the resultant transposon (UPATrap-VC 1, 2, and 3); and (3) by removing the IRES sequence in the *NeoR* cassette. Removing the IRES, which suppresses the nonsense mediated decay of fusion transcripts, biases the selection of resistance towards the integration of the transposon in the 3′ of the captured genes (see Methods).

To validate the gene trap ability of these modified transposons, we also generated an UPATrap-GFP version (full-length GFP in place of the VC), and cotransfected this with transposase-expressing plasmid into both HeLa and CAL51 tumor cells. After neomycin selection, we found that 19% of CAL51 neomycin-resistant (Neo^r^) clones and 43% of HeLa Neo^r^ clones were GFP-positive. By isolating individual colonies from GFP-positive CAL51 cells, we found that each colony exhibited a different localization of the GFP signal (Fig. 3c), suggesting that the transposon had trapped different genes in each case. On the basis of these successful validation experiments, we generated gene trapped CAL51 cell libraries (i.e., populations of cells with the gene trap in different genes in different cells) using UPATrap-VC 1, 2, and 3 (Fig. 3a, b). We selected CAL51 cells in this instance as these cells have a diploid genome and an absence of large-scale genomic rearrangements. Ten million cells were electroporated with transposase plasmid and UPATrap-VC 1, 2, and 3 using a limiting dilution of transposon DNA to maximize the likelihood of a single transposon integration event per cell (Supplementary Fig. 3b, c, d)^22^, generating ~50,000 gene-trapping events after neomycin selection. We divided this library into six sub-libraries and then transfected each sub-library with either PBZ-GFP, PBZ-VN, or PBZ-4A-VN sensors (Fig. 3a(ii)). The PBZ-GFP construct shows the efficiency of the electroporation, which was typically in the range of 60-65% (Supplementary Fig. 3e). After culturing cells for 48 h, GFP-positive cells were isolated by FACS (Fig. 3a (iii)). Compared to PBZ-GFP, introduction of PBZ-VN caused < 0.1% of cells to become GFP-positive, as expected for a probe only detecting VC tagged PARylated proteins. After isolating genomic DNA (gDNA) from GFP-positive cells, we identified gene targeting events using an optimized non-restrictive linear amplification PCR method (nrLAM-PCR)^23^. This method amplifies the genomic region adjacent to each UPATrap-VC transposon insertion site, which was then sequenced using Ion Torrent sequencing. This PCR method was specific, as PCR products were only obtained from UPATrap-VC transposed cells and not from non-transposed cells (Supplementary Fig. 3f, g). We developed a pipeline to align and annotate the sequenced reads as a means to identify UPATrap-VC insertion sites (see Methods). Each gDNA was amplified and sequenced in triplicate, resulting in a highly specific pattern of distribution of the sequencing reads (Supplementary Fig. 3h, i). We found that the number of unique DNA reads generated by triplicate amplification/sequencing procedures for each UPATrap-VC insertion site to be highly correlated (Spearman’s correlation *r*_s_ > 0.98, Fig. 3d). In total, we identified 400 UPATrap-VC insertion sites in the GFP-positive cells; 50 of these genomic sites were represented with a high number of unique reads ( > 30 reads/site), with the rest forming a long-tail distribution in terms of read depth (Fig. 3e). From these 50, we filtered out genomic loci identified in the PBZ-4A-VN screening arm as likely false positives, as well as those UPATrap-VC insertion sites unlikely to form genuine gene-VC, e.g., those integrations in gene deserts. The remaining 20 UPATrap-VC insertion sites were located in 17 genes, with three genes showing two independent integration events: *NPM1* (nucleophosmin, B23), *CTIF* (cap-binding complex dependent translation initiation factor) and CCDC171 (Coiled-coil domain containing 171) (Fig. 3f and Table 1). Given the low transposon/cell number ratio used, these multiple insertion events in *NPM1*, *CTIF*, and *CCDC171* originated in different cells and, therefore, represented independently occurring events. Due to the complexity of the screen, and to the depth of sequencing that can be achieved with the nrLAM-PCR, we believe that the screen was conducted in under-saturating conditions. It is likely that we show a sampling of the PARylome and that further iterations of the screen would be necessary to achieve saturation.

**Table 1:** A summary of the Tol2 integration sites identified in the PARylation screens

Chr	Strand orientation	Insertion site (genomic position)	Median_read_sample	Nr Insertion sites	Gene	Gene ID	Truncation (N-terminal AA)	Protein length (AA)
5	+	170827447	502	2	*NPM1*	ENSG00000181163	195	259
5	+	170823418	321	2	*NPM1*	ENSG00000181163	174	259
18	+	46304668	1131	2	*CTIF*	ENSG00000134030	459	600
18	+	46366092	777	2	*CTIF*	ENSG00000134030	551	600
9	+	15888921	471	2	*CCDC171*	ENSG00000164989	1200	1326
9	+	15887412	361	2	*CCDC171*	ENSG00000164989	1200	1326
1	+	95306653	972	1	*SLC44A3*	ENSG00000143036	253	653
8	-	119229899	612	1	*SAMD12*	ENSG00000177570	155	161
6	+	25600895	572	1	*LRRC16A*	ENSG00000079691	1039	1371
5	-	76998018	420	1	*TBCA*	ENSG00000171530	55	108
19	+	39122699	377.5	1	*EIF3K*	ENSG00000178982	119	218
3	-	30786048	376	1	*GADL1*	ENSG00000144644	464	521
19	-	40368616	339.5	1	*FCGBP*	ENSG00000090920	4157	5405
9	+	130443026	313.5	1	*STXBP1*	ENSG00000136854	515	594
3	+	142745673	166	1	*U2SURP*	ENSG00000163714	426	1029
19	+	10797868	144	1	*ILF3*	ENSG00000129351	690	702
2	-	45729067	80	1	*SRBD1*	ENSG00000068784	624	995
3	+	29904702	43.5	1	*RBMS3*	ENSG00000144642	212	433
3	+	155641886	38	1	*GMPS*	ENSG00000163655	478	693
4	+	71665115	22	1	*RUFY3*	ENSG00000018189	520	620

The position of the integration, the host gene information, the number of the unique-length reads obtained by the nrLAM-PCR, and the length of the N-terminally truncated fragment for each protein are shown for each insertion site

One of these three genes identified by independent integration events, *NPM1*, encodes a known PARylated protein that resides, together with PARP1/2, in the nucleoli of cells^9, 24, 25^. We found that the NPM1-VC + PBZ-VN GFP signal had a precise nucleolar localization, which partially coincided with endogenous NPM1 as shown by antibody staining (Fig. 3g). The identification of this bona fide PARylated protein gave us confidence that we have identified genuine PARylation targets in the screen. We also generated VC sensors for an additional ten candidate PARylated target proteins and examined the cellular localization with the PBZ-VN. In each case, we observed a specific subcellular localization pattern (Fig. 3h). We validated these localization patterns in the case of CTIF (Fig. 3g and the rest of this study) and ILF3 (Supplementary Fig. 2d) by antibody co-staining, which showed partial overlap with between the protein and the biosensor. It is important to point out that we have attempted to co-localize the biosensor signal for a subset of these genes with a staining with PAR-binding reagent (Millipore). As shown in Supplementary Fig. 2d, in five examined cases we failed to observe co-staining (with an exception of CTIF where a mild co-localization is observed; this point is addressed below). Crucially, two of these proteins (TERF1 and GLUT4) are validated PARylation targets, which this staining failed to confirm. This shows that staining with the PAR reagent is not a reliable way to validate targets, and that the biosensor has the potential to achieve higher sensitivity.

We chose to further characterize CTIF as it was identified with multiple transposon integration sites and its biosensor exhibited a specific, perinuclear localization (Fig. 3g and Fig. 4).

### CTIF is a TNKS-dependent PARylation target

We identified two independent integration sites in the last two introns of the *CTIF* gene, generating two C-terminal truncations as shown in Fig. 4a, b, c. Other than its role in CBP80/20-dependent translation and nonsense mediated RNA decay^26^, very little is understood about the function of CTIF. Examination of the CTIF-VC + PBZ-VN (or CHFR-PBZ-VN) sensor signal suggested that CTIF PARylation was localized to a cluster of perinuclear granules (Fig. 4d). Importantly, this localization was not observed when PBZ-4A-VN sensor was used (Supplementary Fig. 4a). The expression of a VN-only fragment, showed a diffuse cytoplasmic localization (Supplementary Fig. 4a), similar to a full-length CTIF-GFP sensor (Fig. 4e), suggesting that CTIF was also localized to the cytoplasm, similar to previous observations^26^. However, PARylation of CTIF, as detected by CTIF-VC + PBZ-VN sensors, occurred predominantly in the centrosomal area of the cell (Fig. 4e). We validated the centrosomal location by the expression of centrosomal markers (CETN2-GFP or Cep170-GFP) or staining for centrosomal markers (CETN3 and gamma-Tubulin) (Fig. 4e). Interestingly, endogenous CTIF showed enrichment only at the daughter centriole—it co-localizes with one of the CETN2-GFP marked centrioles, but is completely excluded from the mother centriole, marked by Cep170-GFP (Fig. 4e, f and Supplementary Fig. 4c). This shows that CTIF has a bona fide centrosome targeting. This is further substantiated by CTIF-GFP, which shows a broad cytoplasmic distribution with enrichment at the centrosome (Fig. 4e). In contrast, CTIF-VC + PBZ-VN biosensor shows strong centrosomal enrichment in an area surrounding the centrosomal markers used (Fig. 4e).

One of the transposon insertions in *CTIF* was predicted to fuse VC to a truncated CTIF protein comprising the N-terminal 511 out of the 600 amino acids of the full-length protein (CTIF 1-511). We generated a CTIF 1-511-VC sensor, which when combined with PBZ-VN, generated an identical GFP localization pattern to full-length CTIF-VC + PBZ-VN (Supplementary Fig. 4b). In order to identify which PARP superfamily member is responsible for the CTIF biosensor behavior, we exposed cells to either olaparib (a PARP1/2 inhibitor) or ICR-TNKS-001 (a tankyrase inhibitor^8^), and assessed the CTIF-VC + PBZ-VN signal. While the signal was unaffected by olaparib exposure, its intensity and cellular distribution was largely suppressed by the tankyrase inhibitor (Fig. 4g). Tankyrase inhibition did not abolish the ability of CTIF to bind to the centrosome (Supplementary Fig. 4c), but rather abolished the formation of the biosensor signal in the broader area surrounding the centrioles. Furthermore, RNA interference-mediated silencing of *TNKS* and *TNKS2* led to decreased CTIF-VC + PBZ-VN biosensor signal without affecting the total CTIF level (Fig. 4h, i). CTIF-GFP immunoprecipitation revealed that CTIF is directly PARylated, and that this PARylation was suppressed by tankyrase inhibition (Fig. 4j and Supplementary Fig. 4f). Probing the same blots for the presence of tankyrase failed to identify an interaction. Tankyrase binds its targets by recognizing a canonical motif^27^. By this definition CTIF posses two motifs in its N-terminal domain, albeit with a suboptimal sequence (lacking the critical arginine residue at position + 1) (Supplementary Fig. 4d). We generated a CTIF mutant with the critical amino acids at the + 1 and + 6 positions in each motif replaced by alanine and assessed its cellular localization; no significant change in the intensity or localization of the biosensor signal of the mutant was observed when compared to wild-type CTIF (Supplementary Fig. 4e), suggesting that these two motifs are not genuine tankyrase recognition sequences. Yet, by generating an allelic series of CTIF-VC sensors with different CTIF deletion events, we found that the N-terminal 100 amino acids of CTIF were sufficient for the localization of CTIF-VC + PBZ-VN to the centrosome, while the C-terminal domain interacts with a pool of cytoplasmic granules (Fig. 4k and Supplementary Fig. 4g). Taken together, these data suggested that CTIF is a direct target of tankyrase-mediated PARylation, but its recognition may be mediated through interactions with another proteins, potentially located in the N-terminal portion of the protein.

### CTIF affects the distribution of centrosomal satellites

To understand the nature of CTIF PARylation, we exploited the ability of a PAR biosensor to monitor the temporal and subcellular localization changes in the passage of a cell cycle. We introduced CTIF-VC + PBZ-VN or CTIF-GFP biosensors into H2B-Cherry expressing HeLa cells and imaged them over one cell cycle (Fig. 5a). While CTIF-GFP showed a largely homogeneous cytoplasmic distribution throughout the cell cycle, the CTIF-VC + PBZ-VN GFP signal was clustered in granular structures around the centrosome during interphase (Fig. 5a). As cells progressed towards mitosis the biosensor intensity increased and coalesced at the centrosome in G2 (Supplementary Fig. 5a); in mitosis the signal clearly segregated along with the two centrosomes. These granular structures were transported towards the centrosome in a microtubule-dependent manner as exposure of cells to the microtubule depolymerizing agent (nocodazole) led to their reversible cytoplasmic dispersal; a microtubule-stabilizing agent (paclitaxel) had no such effect (Fig. 5b).

Tankyrase is a known regulator of centrosome behavior^5, 28^, and our data suggested that it PARylaes CTIF. The dynamic pattern of CTIF PARylation was reminiscent of the behavior of the centrosomal satellites^29, 30^. This prompted us to further investigate localization of the CTIF biosensors with respect to various additional centrosomal markers (Fig. 5c). We found that the CTIF-VC + PBZ-VN surrounded the inner centrosomal markers CETN2, CETN3, PCNT, and gamma-tubulin (Fig. 4e and Fig. 5d). It also occupied the centrosomal area together with various centrosomal satellite markers (PCM1, Cep131/Azi1, Cep290, and BBS4, Fig. 5d), although not co-localizing with them. Tankyrase co-localized with the CTIF-VC + PBZ-VN biosensor signal (Fig. 5e), supporting our previous data that suggested that CTIF might be a tankyrase target protein.

To investigate the functional effect CTIF might have on centrosomal proteins, we depleted CTIF by RNA interference (RNAi). While *CTIF* RNAi did not obviously influence the localization of inner centrosomal markers (e.g., PCNT, Fig. 5f, h), certain centrosomal satellite proteins (Cep131/Azi1, Cep290, and BBS4) became less localized (quantification for Cep131/Azi1 is shown in Fig. 5f, g). Interestingly, other satellite markers (e.g., PCM1 and OFD1) seemed unaffected (Supplementary Fig. 5b; phenotypes are summarized in Table 2). Tankyrase depletion had a similar, although less pronounced effect, upon the same subset of markers (Fig. 5f, g). Taken together with our earlier observations, this data suggested that CTIF PARylation, most likely via tankyrases, is associated with centrosomes, and that this tankyrase-CTIF axis might play a role in the localization or recruitment of centrosomal satellite proteins.

**Table 2:** A summary of the CTIF and tankyrase (TNKS + TNKS2) depletion phenotypes on various centrosomal markers

Marker protein	CETN3	PCNT	PCM1	BBS4	Cep290	Cep131 Azi1	OFD1	TNKS TNKS2	CTIF-VC WT-VN
Localization	Centrioles	Centrioles PCM	PCM CS	CS	CS	CS	CS		
RNAi									
siCon	**+**	**+**	**+**	**+**	**+**	**+**	**+**	**+**	**+**
siTNKS + siTNKS2	**+**	**+**	**+**	**+/−**	**+/−**	**+/−**	**+**	**−**	**−**
siCTIF	**+**	**+**	**+**	**−**	**−**	**−**	**+**	**+**	**−**

Various centrosomal markers were immunodetected (as in Fig. 5d) after RNAi-mediated depletion of either CTIF, or TNKS + TNKS2 in either HeLa, or CAL51 cells. “ **+** ” denotes localization similar control depleted cells, “ **+** **/**−” denotes diminished centrosomal localization, “**-**” denotes absent or severely diminished centrosomal localization

## Discussion

In this study, we describe a biosensor-based approach to identify PARylation events and targets. The system we describe possesses several advantageous qualities. The ability to detect PARylation without the requirement to lyse cells facilitates the detection of steady state PARylation in living cells but also temporal and spatial changes in PARylation. These sensors can be integrated into a genetic screening systems, including the transposon-based system described here, enabling the detection of novel PARylation related events. Our data suggest that CTIF is PARylated in the proximity of the centrosome in a tankyrase-dependent manner. Subsequent work is necessary to investigate CTIF’s precise role at the centrosome. Intriguingly, CTIF localizes to the growing daughter centriole where localized RNA translation could be associated with the translation of a subset of necessary proteins, similarly to what was previously shown in the case of OFD1 (ref.^31^).

There will be caveats associated with the PAR-biosensors, e.g., protein tagging that may alter normal cellular behavior. Importantly, although we have not observed gross aggregation of tagged proteins a major concern remains the level of tagged protein expression, which may cause artefactual phenotypes in the biosensor system. There exists the possibility that the PAR-binding may stabilize PARylation events to some extent. This is advantageous in terms of detecting transient PARylation events, but might also be problematic if it interferes with normal molecular processes. In each case, the observed phenotypes need to be validated by orthogonal methods.

Other methods of gene perturbation, e.g., CRISPR-Cas9-mediated gene tagging (e.g., CRISPaint^32^) or gene mutagenesis (e.g., CRISPR tiling arrays^13^) could be integrated into experimental workflows that use PAR biosensors. The PBZ-based biosensors recognize a specific aspect of the PAR chains (the α(1-2) O-glycosidic bond between the ADP-ribose units); other PAR binding domains (e.g., macrodomains or WWE motifs) recognize different aspects of the PAR chain^33^. We envisage that a range of biosensors can be created that report on different facets of PAR biology. Finally, PAR biosensors could be adapted to capture PARylation events in live animals, which lead to better understanding of the effects of PARP inhibitors in different tissues in vivo.

## Methods

### Cells

CAL51 and HeLa cells were grown in DMEM (Gibco) supplemented with 10% FBS, penicillin-streptomycin (ThermoFisher). CAL51 *PARP1*^–/–^ cells were generated by GE Dharmacon Edit-R Gene Engineering System, by transfecting with 1 μg Edit-R CRISPR-Cas9 nuclease expression plasmid mixed with 2.5 μl of 20 μM *PARP1* crRNA (GAC CAC GAC ACC CAA CCG GAG UUU UAG AGC UAU GCU GUU UUG,) and 2.5 μl of tracrRNA (20 μM), using Lipofectamine 3000 reagent according to the manufacturer’s instructions (Life Technologies). Three days post transfection, cells were selected in 100 nM talazoparib for 5 days, and surviving cells were FACS sorted to isolate single clones. Biallelic genome modification was confirmed by Sanger sequencing. HeLa cells expressing PARP1-LAP construct have been previously described^34^.

### Constructs

The PAR biosensors are based on the APLF amino acids 371–451 or CHFR amino acids 407–665 sequence respectively, which were synthesized and cloned into pEGFP-N1 (Clontech), the *Eco*RI/*Kpn*I site of pBiFC-VN173 (Addgene #22010) and pBiFC-VC155 (Addgene #22011, ref.^35^), and pcDNA3-mRuby2 (Addgene #40260, ref.^36^). In the APLF-based biosensor, Cys-to-Ala mutations were introduced in C379, C385, C421, and C427 to generate the 4A versions of the above constructs. Full-length FLAG-PARP1 was cloned in pEGFP-N1 vector. DNA-binding deficient (p.delM43F44I^13^) and catalytic-deficient mutant (E988K) were introduced with site-directed mutagenesis. Hits identified in the PARylation screen were amplified from a cDNA library and cloned in the pBiFC-VC155 vector. Full-length *CTIF* was amplified from a cDNA library and cloned in pEGFP-N1 or pBiFC-VC155 vector. CTIFm2 mutant, carrying the following substitutions–K44A, G49A, L150A and G155A, was produced by gene synthesis and cloned into the pBiFC-VC155 vector. UPATrap-VC/GFP vectors are based on the UPATrap technology^21^. The IRES-GFP moiety of UPATrap-Tmat vectors 1, 2, and 8 (Genbank accession numbers AB673346, AB673347, AB673353) was replaced by HA-VC155 or HA-EGFP; the HA sequence starts 15 base pairs downstream of the SA and lacks an ATG codon. To cover the three possible reading frames, three vectors were created, which have 0, 1, or 2C bases in front of the HA sequence. The IRES from the splice donor (SD) cassette was excised in order to bias the selection of integration sites towards the last introns. CETN2-GFP (#41147) and Cep170-GFP (#41150) expressing constructs were acquired from Addgene.

### Antibodies

GFP (Roche, 11814460001; 1:2000), PAR 10H (Trevigen, 4335-AMC-050; 1:200), PAR binder (Millipore, MABE1031, 1:1000), CTIF (Sigma-Aldrich, HPA016865-100UL; 1:1000), TERF1 (Abcam, ab10579; 1:1000), GLUT4 (Abcam, ab654; 1:1000), ILF3 (Abcam, ab92355; 1:1000), NPM1 (Abcam, ab10530; 1:1000), CETN3 (Abnova, H00001070-M01; 1:500), PCNT (Atlas antibodies, HPA016820; 1:500), PCM1 (Cambridge bioscience, A301-149A; 1:500), gamma-tubulin (Sigma-Aldrich, T6557-100UL; 1:500), Azi1/Cep131 (Abcam, ab84864; 1:500), Cep290 (Abcam, ab84870; 1:500), BBS4 (Proteintech, 12766-1-AP; 1:500), OFD1 (Proteintech, 22851-1-AP; 1:500), TNKS1/2 (Santa Cruz, sc-8337; 1:1000), alpha-tubulin (Sigma-Aldrich, T9026; 1:10000), beta-actin (Sigma-Aldrich, A1978; 1:10000).

### Reagents

Olaparib (Selleckchem), ICR-TNKS-001 (ref.^8^), Nocodazole (Sigma, M1404-10MG), Paclitaxel, H_2_O_2_ (Sigma, 216763-100 ML), G418 (Sigma, G8168-100ML).

### siRNA

The following siRNAs were provided by GE Healthcare *siCTIF* (M-021020-01-0005), *siTNKS* (M-004740-01-0005), *siTNKS2* (M-004741-01-0005).

### Transfection

DNA constructs were transfected with Lipofectamine 2000 (Life Technologies) according to manufacturer instructions. Typically, for a 24-well plate a mix of 175 ng of gene-VC and 25 ng sensor-VN was transfected. For siRNA transfection Lipofectamine RNAiMax was used (Life Technologies) according to manufacturer's instructions.

### Immunoprecipitation and western blotting

Immunoprecipitation was typically carried out from close-to-confluence 10-cm dishes transfected in advance with appropriate construct. Cells were washed with PBS, collected and lysed in Net-N buffer (50 mM TrisHCl pH7.5, 1 mM EDTA, 0.5% IGEPAL CA-630, 1 mM DTT, protease inhibitors (Sigma, cOmplete mini), sonicated and cleared by centrifugation. 0.5–1 mg total protein extract was incubated with GFP_Trap beads (Chromotek) for 1 h at 4 °C with rotation. Beads were washed five times with lysate buffer and proteins were eluted by heat denaturation in loading dye. Samples were resolved on NuPAGE protein gels (ThermoFisher), transferred to nitrocellulose membrane and blocked in 5% milk. Anti-PAR (1:2000) and anti-GFP (1:2000) were incubated at 4 °C overnight. Proteins were detected and quantified on the Odyssey Fc imaging system (LiCor).

### Cellular libraries construction and screening

In all, 1 × 10^7^ CAL51 cells were electroporated with 30 ng UPATrap-VC (each open reading frame) and 1400 ng Tol2 transposase-expressing plasmid. They were split and kept as six independent cellular libraries. The cells were selected with 0.8 mg/ml G418 for 10 days. Colony formation assay showed that each cellular library contained ~5000 independent clones. Libraries were expanded to a representation of 1000 cell/clone and frozen down. For the PARylation screen, each library was divided into three identical aliquots (each aliquot had 5 × 10^6^ cells, to ensure ~1000 cells representation for each of the 5000 VC-gene trap events). The first aliquot was electroporated with a wild-type (PBZ-GFP) biosensor expression plasmid to monitor the efficiency of electroporation, which was 60%. The second aliquot was electroporated with a wild-type (PBZ-VN) biosensor expression plasmid and the third aliquot was electroporated with a mutant (PBZ-4A-VN) VN linked biosensor. After 48 h, the GFP-positive fraction from the PBZ-VN and PBZ-4A-VN aliquots ( < 0.1 % of each library) was isolated by FACS. These cells were expanded and aliquots were frozen and gDNA prepared (Blood and Tissue kit, Qiagen).

### nrLAM protocol

nrLAM-PCR protocol was adapted and optimized from ref.^23^ as follows. For each gDNA sample three independent reactions were run. Fifty microliters reactions, containing 1.25 U Taq (NEB), 1 μg gDNA, 0.5 μl 0.17 μM biotin-SPL1 primer, and 2 μl 0.5 mM dNTPs, were cycled—95 °C/2’, 50 × (95 °C/45”, 58 °C/45”, 72 °C/10”). After one run, 1.25 U Taq was added and the PCR program repeated. Biotinylated products were collected on streptavidin beads (Life Technologies 11205D), washed, and re-suspended in a ligation mastermix, containing 25% w/v PEG8000 (Sigma 89510-250G-F), 1 μM ssAdapter, 1 mM Co(NH_2_)_6_Cl_3_ (Sigma H7891-5G), 1 × T4 ligation buffer and 20 U T4 ligase (NEB), for 16 h at 25 °C and 300 rpm shaking. Reactions were diluted with 90 μl water, beads were collected and washed with 100 μl water. Finally they were re-suspended in 25 μl water and 5 μl were used in a 50 μl Q5 (NEB) PCR reaction with 0.2 μM SPL1 and 0.2 μM HmSp1 primers—98 °C/30”, 20 × (98 °C/20”, 70 °C/20”, 72 °C/1’), 72 °C/2’. One microliter of this PCR reaction was used as a template in a subsequent reaction: 50 μl Q5 PCR reaction with 0.2 μM P1trunc and 0.2 μM IonTorrent_index primers—98 °C/30”, 10 × (98 °C/10”, 61 °C/10”, 72 °C/1’), 10 × (98 °C/10”, 69 °C/10”, 72 °C/1’), 72 °C/2’. PCR products were obtained only from Tol2-containing gDNA. DNA libraries were purified with a PCR purification kit (Qiagen) and sequenced on Ion Torrent PGM 318 chip, 400 flow.

Biotin-SPL1 – 5′CATGCATCATATCCATCGCAATCGCATCC

ssAdapter – 5’ GATCACCGACTGCCCATAGAGAGGGGTCTCTCCTAGCAACGGTTACTCTTCG (NB: 5′-P, phosphorthioate last C-G bond, 3′-C3 blocking group)

SPL1 – 5′CATGCATCATATCCATCGCAATCGCATCC

HmSp1 – 5′CGAAGAGTAACCGTTGCTAGGAGAGACC

P1trunc – 5′CCTCTCTATGGGCAGTCGGTGATC

AdapterindexTol2-5′CCATCTCATCCCTGCGTGTCTCCGACTCAGN_10_GATTTTGAGTACTTTTTACACCTCTG

### Data analysis

The data analysis pipeline is online in a diagram in Supplementary Fig. 6. Briefly, fastq files were obtained from Ion Torrent sequencing. The Tol2 sequence (5′TTTGAGTACTTTTTACACCTCTG) was removed from all the reads by cutadapt-1.4.2 and they were further aligned to the human genome GRCh37, using bwa-0.7.9a^37^. From the alignment bam/bed files were generated. Using bedtools^38^ master blocks were generated, covering the coordinates of any overlapping bed across all sequenced samples. PCR duplicates were removed from the bed files and only unique-length reads were counted. Each bed file was intersected with the master block and with gene annotation bed files. In this way, one generates a unique integration site identifier (master block) for which unique reads can be counted for each sample. Finally, the unique-length read count information was intersected across samples. This generated the initial hit list of integration sites with their counts.

The initial hit list was subjected to the following filtering criteria. Firstly, integration sites with less than three unique-length reads were discarded. Secondly, every authentic integration site should produce a stacked pyramidal arrangement of reads that have to be co-oriented with the direction of transcription of the host gene, e.g., Supplementary Fig. 3h, i. Hence, anti-oriented sites were discarded. The sites in the resulting list were further filtered based on the following criteria: discarded were sites found in the negative control (PBZ-4A-VN sensor), sites located outside of known genes and sites whose splicing would not lead to the expression of gene-VC fusion (based on the precise location of the integration site within a gene). Furthermore, genes with multiple independently identified integration sites were noted as high-confidence hits.

### Imaging and microirradiation

Cells were seeded on coverslips and transfected with biosensor constructs. After 48 h incubation, the cells were fixed with 1% PFA solution at room temperature for 10 min. Cells were permeabilized with 0.2% Triton X-100 in PBS and blocked with IFF (2% BSA, 1% FBS in PBS). Antibody incubation was carried out in IFF with primary antibody typically in 1:500 dilution; secondary antibody (alexa594-conjugated, ThermoFisher) was used in 1:1000 dilution. Cover slips were mounted on ProLong Gold Antifade (ThermoFisher) and imaged on a Zeiss LSM 780 confocal microscope.

For microirradiation, cells were grown in glass-bottom culture dishes (MaTek, P35G-0.170-14-C) and transfected with required constructs. Twenty-four hours post transfection imaging was done on Andor Revolution system, 60 × water objective with micropoint at 365 nm. Measured were only cell with similar intensity of the GFP signal. The background intensity (in the vicinity of the microirradiation area in the nucleus) was subtracted from that at the microirradiation point and the maximum was normalized to 1. For the FRAP experiments, the same system was used with the following FRAPPA settings–dwell time–60, repeat–10, and laser intensity–6%.

### Data availability

The constructs used in this study are available upon request to the authors or at Addgene (https://www.addgene.org/Chris_Lord/, Plasmid IDs #110646-110653). Transposon integration site sequencing data has been deposited at the European Nucleotide Archive with study number PRJEB26343.

## Electronic supplementary material

Supplementary Information
