# Supplementary material for: Coupling bimolecular PARylation biosensors with genetic screens to identify PARylation targets
Source: Nat Commun. 2018 May 22;9:2016. doi: 10.1038/s41467-018-04466-4 (PMC5964205; doi:10.1038/s41467-018-04466-4)
Supplement: Supplementary file 1 — Supplementary Information [file 41467_2018_4466_MOESM1_ESM.pdf]

Supplementary information

Coupling bimolecular PARylation biosensors with genetic screens to identify PARylation targets

Krastev et al.

Supplementary Figure 1

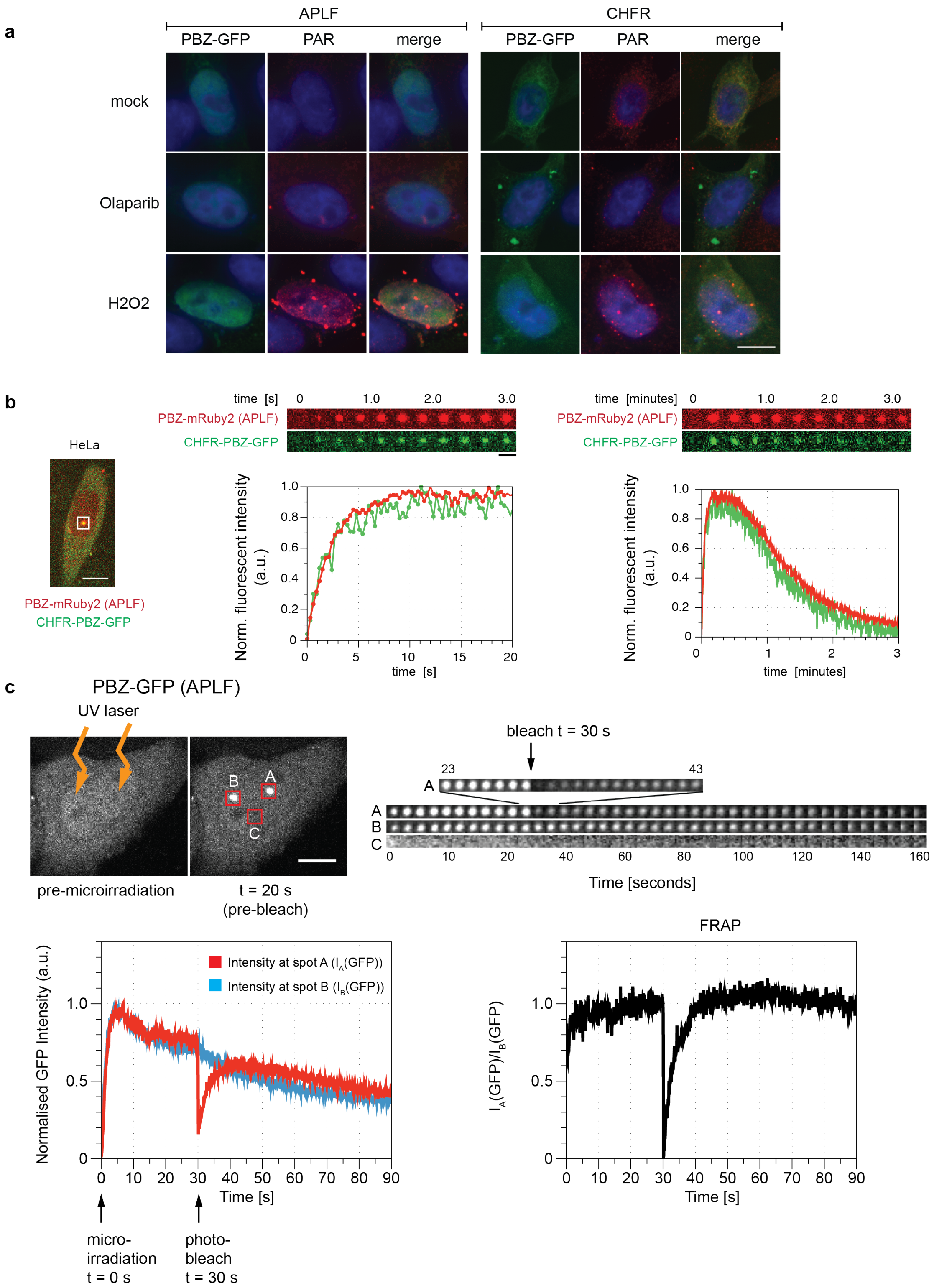

Supplementary Fig. 1. Kinetics of PBZ domains recruitment. (**a**) Exemplary images for the experiments shown in Fig. 1b,c. HeLa cells expressing PBZ-GFP (or CHFR-PBZ-GFP) were exposed to 1 mM H2O2 or the 1 µM olaparib; GFP signal and PAR immunodetection (by use of 10H anti-PAR antibody) were monitored 10 min after exposure. (**b**) APLF- and CHFR- based PBZ domains show similar kinetics of

recruitment to microirradiation induced DNA damage sites. HeLa cells were co-transfected with PBZ-mRuby2 and CHFR-PBZ-GFP constructs and subjected to localised microirradiation. The boxed area, with 2 µm side, is shown as a kymograph above the curves that show the intensity of GFP and mRuby2 recruitment to the site of damage. (**c**) PBZ domain binds reversibly to the sites of damage. HeLa cells were transfected with PBZ-GFP construct. Two spots (A and B) were irradiated simultaneously with an UV lased (t = 0 s). 30 seconds post microirradiation one of the two spots (A) was photobleached (t = 30 s). The recovery of the signal was followed for additional 60 s. The figure shows an exemplary image pre-microirradiation (t = 0 s) and pre-bleaching (t = 20 s). Kymographs of the boxed areas A (bleached), B (unbleached) and C (nucleoplasm) are shown. During the decay phase, spot A is photobleached, with a clear drop of the signal. The signal recovers quickly, reaching the prebleached levels within 10 s. To correct for the concomitant decrease of the signal due to the PAR degradation, we divided the intensity at spot A (I_A) to the one at spot B (I_B). Thus normalized FRAP curve (black) shows the same result – the signal has recovered in under 10 s, indicating a quick exchange rate of the biosensor at the PAR chains. Scale bars represent 5 µm.

Supplementary Figure 2

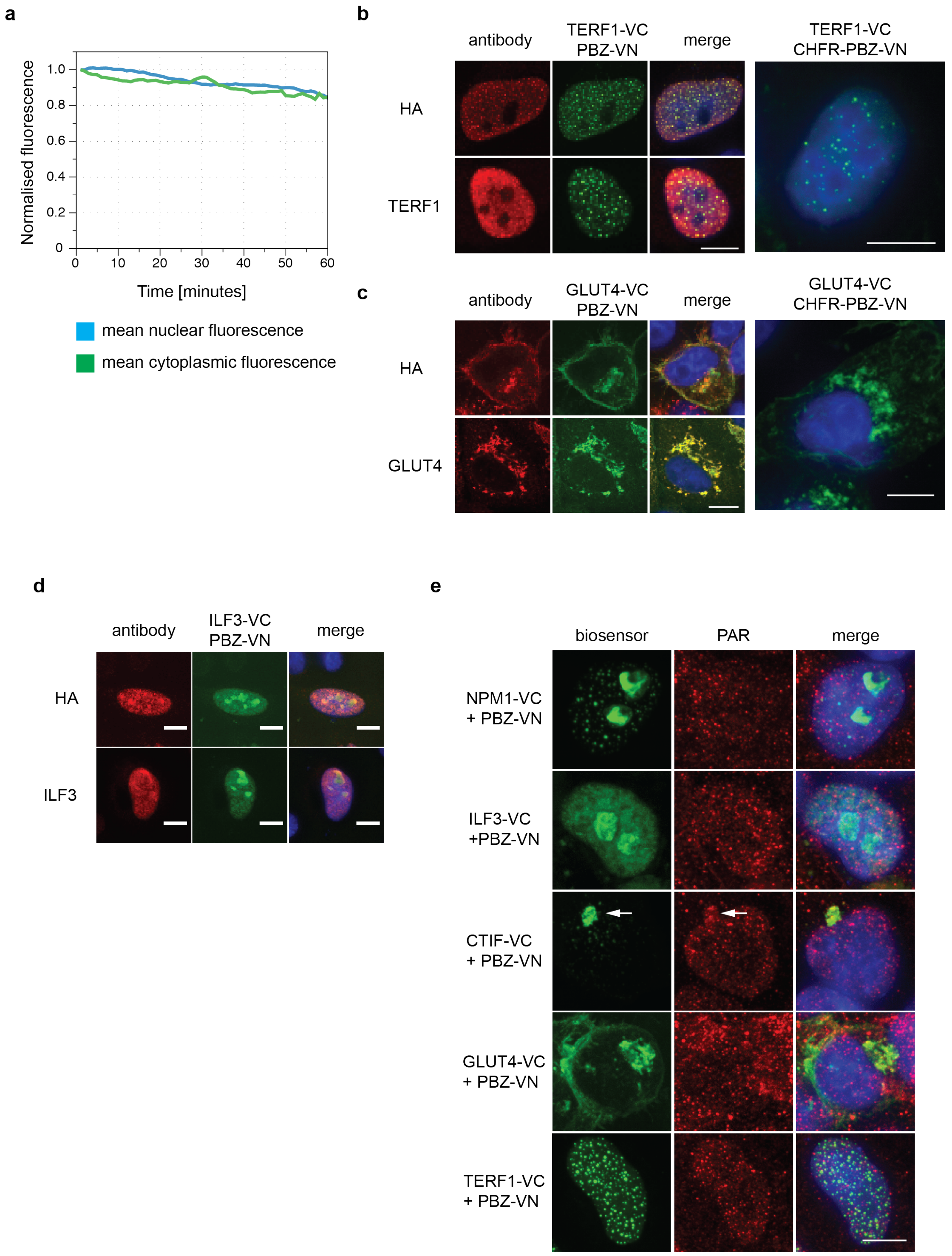

Supplementary Fig. 2. Validation of the biosensor with endogenous antibodies. (**a**) A quantification of the GFP signal decrease in the imaging shown in Fig 2f. The mean nuclear or cytoplasmic GFP fluorescence was measured over the interval of 1 hour imaging (200 ms exposure time, image captured every 60 s). The signal decreased with about 15% over 1 hour, with no significant decrease over the first 10 minutes. (**b**) Comparison of TERF1 PAR biosensor signal detected by immuno-

detection (“antibody detection”), an APLF-derived PBZ PAR biosensor (“PBZ-VN”) or a CHFR-derived PBZ PAR biosensor (“CHFR-PBZ-VN”). HeLa cells were transfected with biosensors as shown and immunostained with either anti-HA (HA sequence is part of the VC construct) or anti-TERF1 antibody. In both cases the antibodies recognize a broader nucleoplasmic protein pool with enrichment of signal at the telomeres. The biosensor shows predominantly telomeric signal, co-localising with the antibody signal. An independent transfection was carried out with TERF1-VC + CHFR-PBZ-VN, indicating that a different PAR binding domain (from CHFR-PBZ-VN) generates biosensor signal similar to the PBZ-VN probe. Scale bars represent 5 μm. (**c**,**d**) Same as in (**b**) replacing TERF1 with GLUT4 or ILF3, respectively. (**e**) Co-localisation of the biosensor signal with PAR detection. HeLa cells were transfected with the indicated biosensor constructs (gene-VC + PBZ-VN), and stained with the PAR-binding reagent (Millipore). A weak co-localisation was observed only in the case of CTIF (indicated with white arrows), which coincides with the centrosomal area of the cell, where PARylation is enriched. In all other cases, no apparent co-localisation is observed. Importantly, even two well-validated PARylated proteins (NPM1 and TERF1) do not show co-localisation with PAR detection under these conditions. This strongly suggests that the biosensor has higher sensitivity to detect specific PARylation *in situ*. Scale bars represent 5 μm.

Supplementary Figure 3

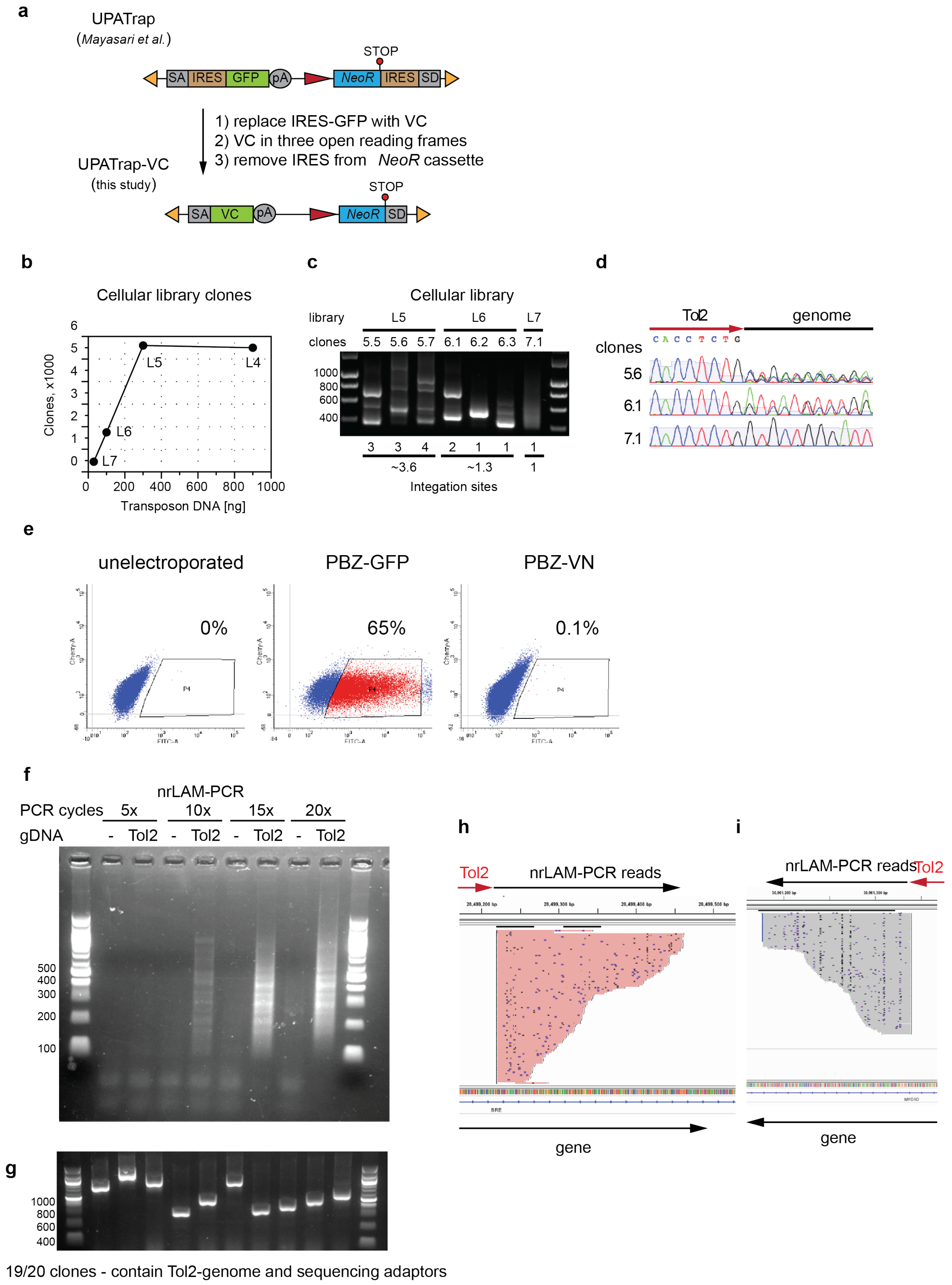

Supplementary Fig. 3. Biosensor-based genetic screen. (**a**) A schematic of the construction of the UPATrap-VC transposon. The UPATrap [1] served as a starting point, to which three changes were introduced: 1) the IRES-GFP cassette was replaced with the VC sequence; 2) the VC sequence was introduced in three open reading frames in a series of three UPATrap vectors; and 3) the IRES from the *NeoR* cassette was removed. (**b**) UPATrap-VC cellular libraries were generated with a

single transposon integration site per cell. CAL51 cells were electroporated with a limiting dilution of UPATrap-VC and excessive amount of Tol2 transposase-expressing plasmid, after which G418-resistant libraries were isolated. Colony formation analysis was used to assess the complexity of the libraries vs the amount of transposon DNA used, where L4 (900 ng), L5 (300 ng), L6 (100 ng) and L7 (30 ng) denote different cellular libraries. (**c**) Single-cell clones were expanded from each library and the number of Tol2 integration sites was assessed with splinkerette PCR. (**d**) Sanger sequencing of the splinkerette PCR products with a Tol2-specific primer confirms the number of integration sites observed at gel, judged by the number of overlapping sequencing traces. (**e**) Cellular library L7 was electroporated with either PBZ-GFP or PBZ-VN expressing plasmids. 65% of the cells were efficiently electroporated, as shown by the PBZ-GFP sample. In comparison, the introduction of the PBZ-VN probe led to the detection of 0.1% GFP+ cells. (**f**) nrLAM-PCR detects products only in Tol2-containing DNA. With increasing the number of PCR cycles, PCR products were observed only in Tol2-containing gDNA. These PCR products were TOPO cloned, individual bacterial colonies were picked and the PCR product was amplified. (**g**) A gel showing 10 individual TOPO clones; Sanger sequencing confirmed the presence of the Tol2, flanked by distinct genomic sequence in 19 out of 20 sequenced clones. (**h**,**i**) An example of the nrLAM-PCR products distribution at two independent Tol2 insertion sites. When aligned to the genome, nrLAM-PCR reads form a characteristic pyramidal stack that is co-oriented with the direction of transcription of the host gene; all reads start at the same location, which is the site of Tol2 integration, and extend into the flanking genomic region.

Supplementary Figure 4

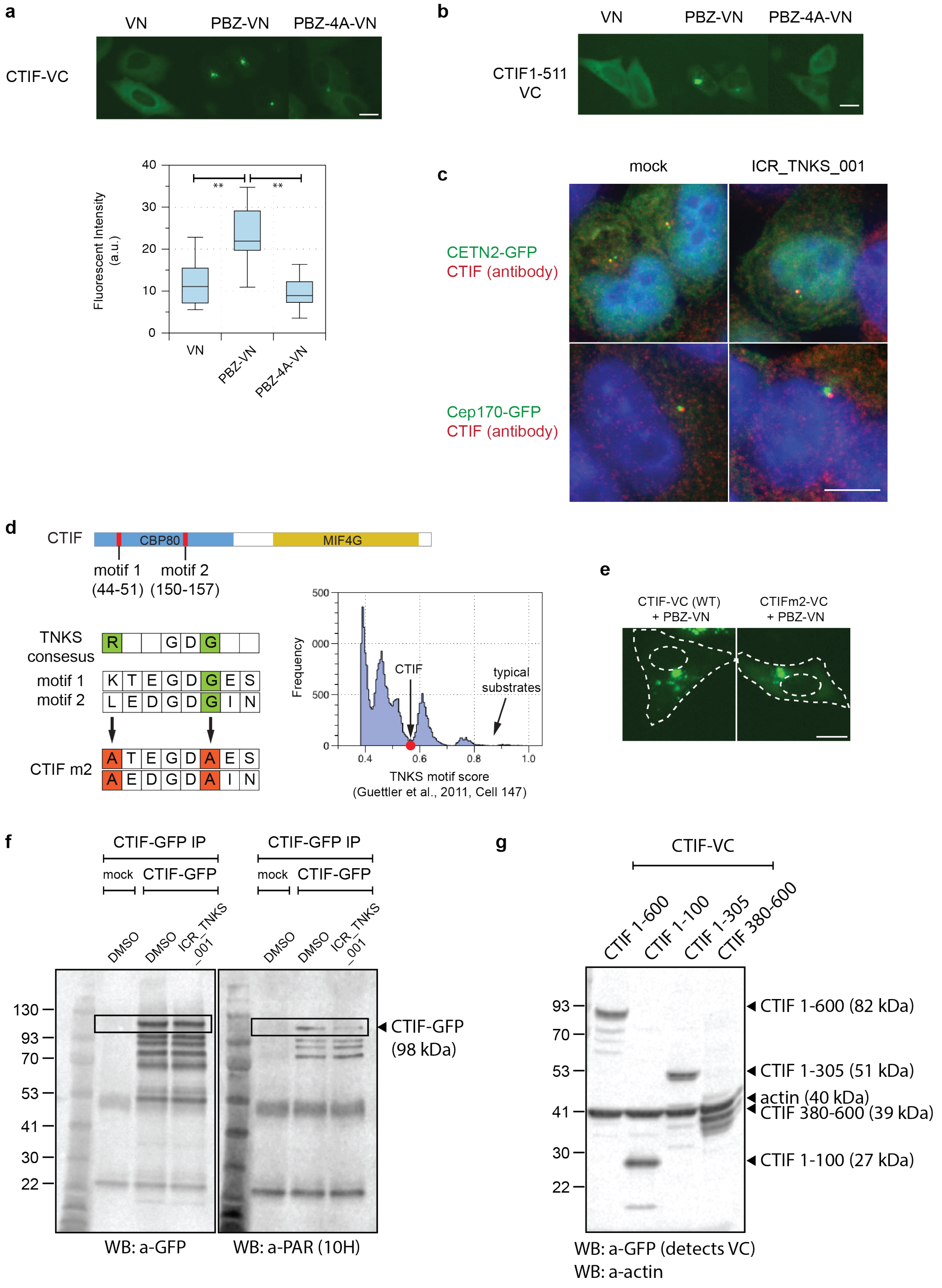

Supplementary Fig. 4. CTIF is a tankyrase-dependent PARylation target. (**a**) Subcellular localisation of CTIF-VC + PBZ-VN biosensor GFP signal in the nuclear proximity visualised by live cell imaging. HeLa cells were transfected with CTIF-VC in combination with VN (no PBZ domain), PBZ-VN or PBZ-4A-VN. Quantification of the mean GFP signal at the centrosome, box plot shows quartiles, Student's *t*-test ** - *p*-

values <0.01. (**b**) As in (**a**) but the full-length CTIF-VC construct was replaced by a truncation construct (expressing amino acids 1-511) that is equivalent to the one isolated by the genetic screen, CTIF1-511-VC. (**c**) CTIF localises to the daughter centriole, irrespective of tankyrase PARylation. HeLa cells were transfected with CETN2-GFP (marks both centrioles) or Cep170-GFP (marks the mother centriole), treated with a tankyrase inhibitor (1 μM ICR_TNKS_001 for 24 h) and stained for the endogenous CTIF. (**d**) CTF tankyrase motifs mutagenesis. Two putative tankyrase recognition motifs were identified *in silico* by Guettler et al., motif 1 (amino acids 44-51) and motif 2 (amino acids 150-157) [2]. In comparison with the canonical binding motif they lack the critical arginine at position 1. Their scores were around 0.55 as defined by the same paper (see histogram), while typical tankyrase substrats display scores >0.8 (albeit axin1 having a score of 0.38). Both motifs were subjected to site-directed mutagenesis: the critical amino acid (highlighted in green in the consensus motif) at position 1 and the glycine residue at position 6 were replaced by alanine substitutions (highlighted in red), generating the CTIFm2 mutant. (**e**) CTIFm2-VC + PBZ-VN showed robust centrosomal signal similar to the wild type CTIF-VC + PBZ-VN biosensor. (**f**) Uncropped scans of the Western blots, shown in Fig. 4j. (**g**) Western blot showing the expression level of the CTIF-VC deletion constructs, shown in Fig. 4k. Scale bars represent 5 μm.

Supplementary Figure 5

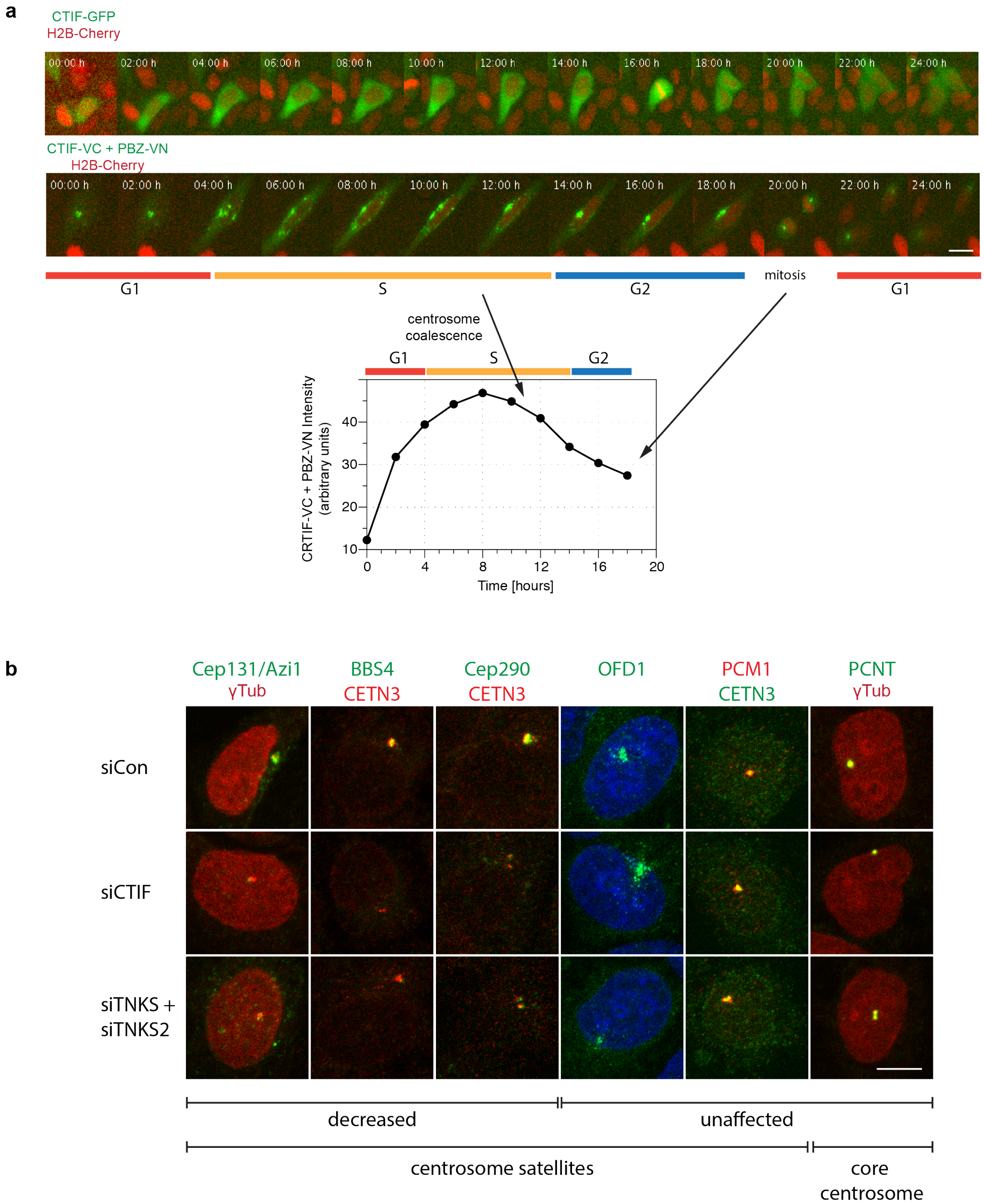

Supplementary Figure 5. CTIF regulates the centrosomal satellites. (**a**) CTIF biosensor distributes with the centrosome during the cell cycle. Expanded kymographs throughout the cell cycle of the constructs shown in Fig. 5a. HeLa cells, expressing H2B-cherry were transfected with CTIF-GFP or (CTIF-VC + PBZ-VN), and imaged in the course of 24 hours. (**b**) CTIF and tankyrase regulate a subset of centrosomal satellite markers. HeLa cells were depleted for CTIF or tankyrase (TNKS + TNKS2), and immunostained for centrosomal markers. Core centrosome (e.g. PCNT) and a subset of centrosomal satellite markers (PCM1 and OFD1) were not altered by this treatment, while other satellite markers (Cep131/Azi1, Cep290 and

BBS4) showed a defect in their centrosomal localisation. This effect is quantified in Table 2. Depending on the origin of the antibody, we used as a centrosome marker either gamma-tubulin or CETN3. Scale bars represent 5 μm.

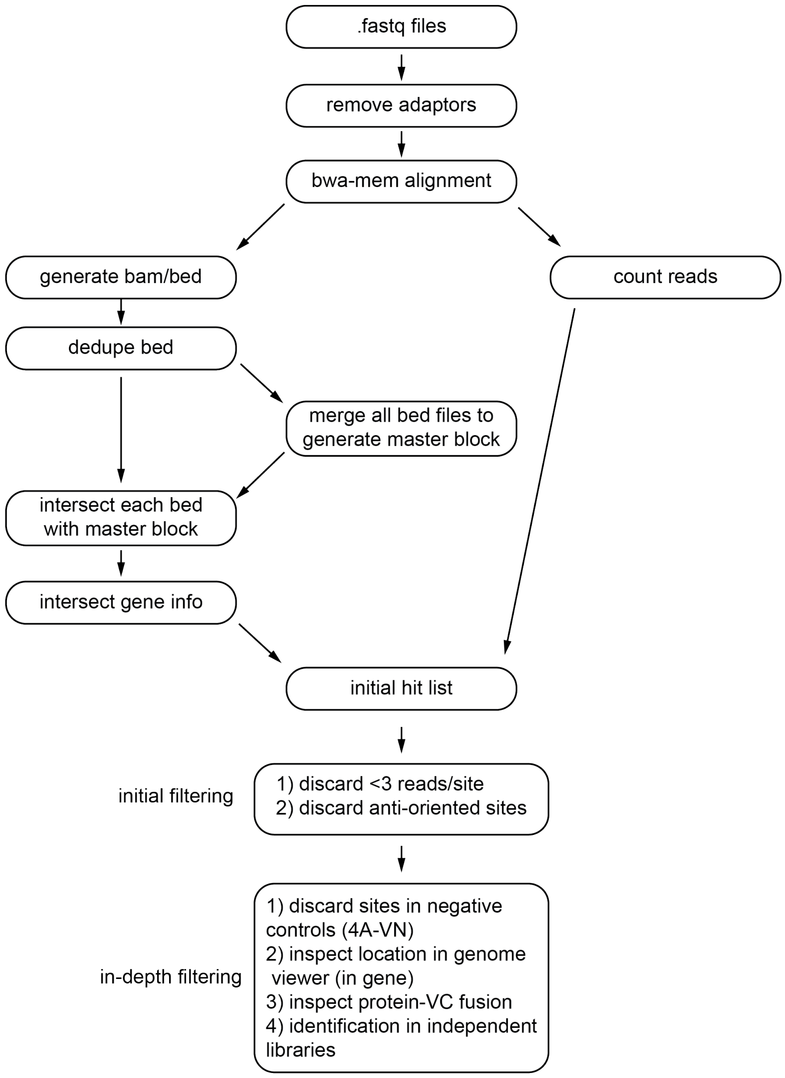

Supplementary Figure 6. Deep sequencing analysis workflow. A diagram representing the pipeline of deep sequencing data analysis as described in Methods.

References

1. Mayasari, N.I. et al. Mixture of differentially tagged Tol2 transposons accelerates conditional disruption of a broad spectrum of genes in mouse embryonic stem cells. *Nucleic Acids Res* **40**, e97 (2012).
2. Guettler, S. et al. Structural basis and sequence rules for substrate recognition by Tankyrase explain the basis for cherubism disease. *Cell* **147**, 1340-1354 (2011).
